# Recurrent epiphora after dacryocystorhinostomy surgery: Structural abnormalities identified with dacryocystography and long term outcomes of revision surgery: Success rates of further surgery following failed dacryocystorhinostomy surgery

**DOI:** 10.1186/s12886-021-01869-8

**Published:** 2021-03-05

**Authors:** Hannah M. Timlin, Swan Kang, Kailun Jiang, Daniel G. Ezra

**Affiliations:** 1 Moorfields Eye Hospital NHS Trust, 162 City Road, London, EC1V2PD UK; 2 Department of Surgery, Division of Ophthalmology, University of Calgary, Calgary, Alberta T2N 1N4 Canada; 3 UCL Institute of Ophthalmology, Bath Street, London, EC1V 9LH UK; 4 NIHR Biomedical Research Centre for Ophthalmology, City Road, London, EC1V 2PD UK

**Keywords:** Dacryocystography, Dacryocystorhinostomy, Nasolacrimal duct obstruction, Lester Jones tube, Epiphora

## Abstract

**Background:**

To investigate the aetiopathology of recurrent epiphora or stickiness after dacryocystorhinostomy (DCR) surgery, identifiable on dacryocystography (DCG), and to assess the success rates of secondary corrective surgeries.

**Methods:**

Consecutive post-DCR DCG images from patients with recurrent symptoms were reviewed between 2012 and 2015.

**Results:**

One hundred fifty-nine eyes of 137 patients were evaluated. Fifty-eight DCGs showed normal postoperative findings, 4 an upper/lower canalicular block, 13 a common canalicular block, 31 a completely closed anastomosis, 50 a narrow anastomosis, and 3 an anastomosis draining into a nasal sinus.

The most successful corrective procedures for each failure category were: Lester Jones Tube (LJT) for a normal post-operative DCG (17/18 success), Sisler trephination with tubes for upper/lower canalicular block (1/2 success), redo-DCR with tube for common canalicular blockage (5/6 success), redo-DCR +/− tube for completely closed anastomosis (12/16 success), LJT followed by redo-DCR +/− tube for narrow surgical anastomosis (1/1 and 17/27 success respectively), and redo-external-DCR with tube for anastomosis into a nasal sinus (1/1 success). Redo-DCR was ineffective in patients who had good post-DCR anatomical patency (22% success).

**Conclusion:**

This is the first study to report success rates of redo-DCR surgery according to anatomical findings confirmed by DCG. The outcome flow diagram help clinicians recommend procedures that are most likely to be successful for their patient’s specific anatomical abnormality. It also provides a visual tool for the shared decision-making process. Notably, symptomatic patients with a normal DCG post DCR are unlikely to benefit from redo-DCR, with a LJT being the recommended next step.

**Supplementary Information:**

The online version contains supplementary material available at 10.1186/s12886-021-01869-8.

## Background

The aim of Dacryocystorhinostomy (DCR) surgery is to form an anastomosis between the lacrimal sac and nasal space facilitating tear drainage. Primary DCR surgery has a published failure rate of 3–13% [1–4]. Although patient numbers are small, failure after DCR surgery presents a difficult management problem. Repeat DCR surgery is less successful than primary surgery with published functional failure rates of 15–22% [5, 6] in redo external DCR and 9–21% [7, 8] in redo endonasal DCR. DCR failure can be due to a range of causes at different anatomical sites along the lacrimal drainage pathway and there is no data available that stratifies the revision surgery success rates based on the type and location of anatomical failure. When approaching revision DCR surgery, it is imperative to determine the cause of failures in order to identify which groups of patients benefit the most and least from different types of surgery. This will allow clinicians to realistically and individually predict surgical success rates and manage patient expectations. Additionally, those with an anatomical failure subtype, which is predicted to have a low redo DCR success rate, could then be offered alternative nasal lacrimal surgery such as Lester Jones Tube or Sisler trephination.

In the authors’ department, dacryocystography (DCG) is routinely performed on patients who report recurrence of symptoms following DCR surgery to visualise the postoperative anatomical flow pathway. Although it is possible to identify the cause of failure based on clinical examination findings, DCG is also useful in identifying the anatomical abnormality of nasolacrimal systems [9, 10] and has been employed in this study to categorise patients by anatomical causes of failure.

The primary aim of this study was to investigate the potential causes of recurrent epiphora and stickiness following DCR surgery, identifiable on DCG imaging. The secondary aim of this study was to establish the outcomes for subsequent revision surgery. This study intends to provide guidance for the decision-making process for patients experiencing failure of DCR surgery by providing surgical success rates of subsequent revision surgery according the underlying cause of failure.

## Materials and methods

This study was conducted in full compliance with the declaration of Helsinki. Permission to perform this study was granted by Moorfields Eye Hospital NHS Foundation Trust Audit committee review board (reference number: CA16/AD/19).

The Moorfields Eye Hospital lacrimal clinic is a tertiary referral center for complex lacrimal disease. Consecutive patients experiencing failure after DCR from 2012 to 2015 were reviewed. A lacrimal consultant reviewed DCG images and identified any DCG abnormalities (DE). Clinical data were collected from electronic and paper hospital records. Symptomatic success after lacrimal surgery was classified as the patient being subjectively satisfied with the outcome and reporting dabbing the eye with a tissue less than once per day, on a typical day (Munk score of 0 or 1).

### Dacryocystography

DCG was performed using a 24G Rabinov Sialography Set filled with Lipiodol Ultra fluid (dye). The lower canaliculus was cannulated to the midpoint. The tubing was taped to the cheek. Video fluoroscopy was performed as the doctor introduced the dye until the point the dye had reached the nasal space or significant reflux was observed. An erect x-ray was then taken 10 min later to determine if there was sequestration of dye within a closed cavity system.

### Statistics

Data was analyzed using Microsoft Excel (Microsoft Office 15.0, 2013, Redmond, Washington).

## Results

### Patient demographics

Over a 4-year period, 160 eyes of 138 patients were identified (Additional file 1). One eye of 1 patient was excluded due to poor quality DCG that precluded interpretation. The cohort was predominantly female (92, 67%). The median age of these patients was 62 years (range 2–90). The majority of patients were of Caucasian (62, 45%), South Asian (33, 24%) or Afro-Caribbean (13, 9%) origin. A further 16 (12%) patients identified ethnically as ‘other’ and 11 (8%) patients were of unknown ethnicity. East Asian and mixed accounted for only 2% of patients (2 and 1, respectively). All patients experienced epiphora with 17 (11%) eyes also having sticky discharge.

One hundred thirty-eight eyes had undergone one previous DCR, 15 eyes had undergone 2 previous DCRs and 3 eyes had undergone 3 previous DCRs.

### DCG findings

**Fig. 1:**
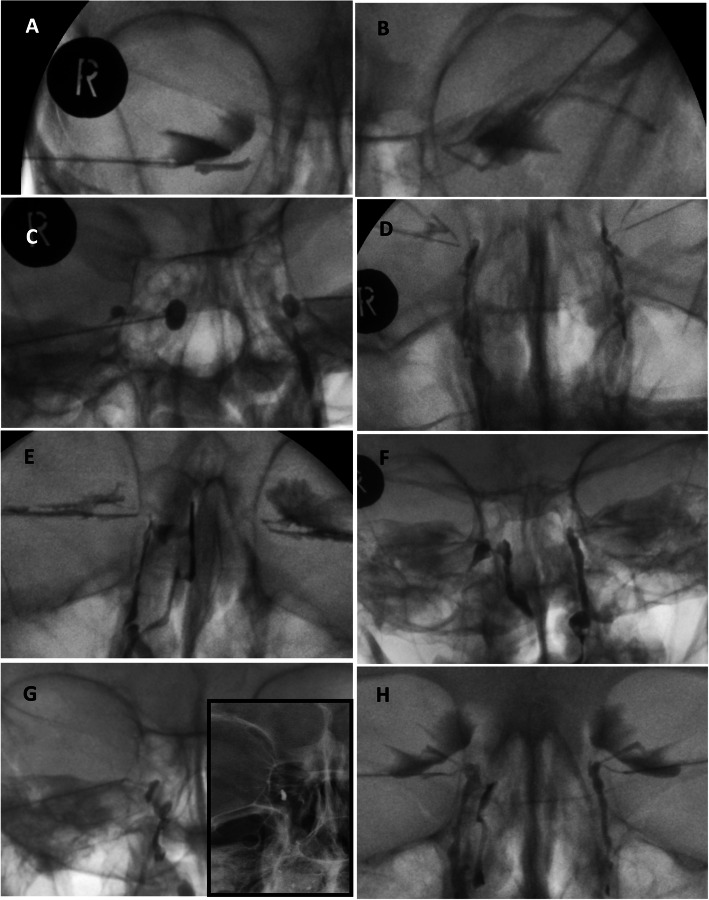
Images showing the different abnormalities seen on DCGs following DCR surgery. **a** right inferior canalicular block, **b** left common canalicular block, **c** right complete surgical anastamosis closure with closed sac remnant, **d** left complete surgical anastamosis closure with flow through the nasolacrimal duct, **e** right narrow anastomosis with narrow flow-stream into the nasal space, **f** right narrow and high anastamosis, **g** right narrow anastamosis with retained dye on erect x-ray, **h** right narrow anastamosis with dye also passing through the nasolacrimal duct

**Fig. 2:**
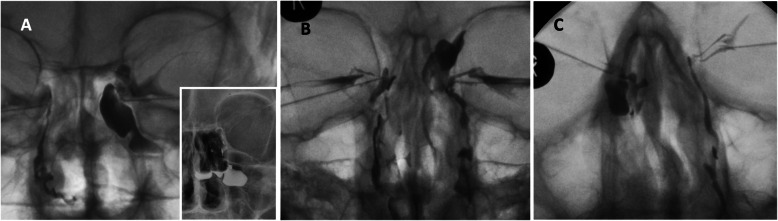
DCG images showing drainage into the nasal sinuses. **a** + **b** left, **c** right. The left, dye filled an ethmoidal air cell as well as passing though the nasolacrimal duct

Upon review, 58 (36.3%) DCGs showed normal postoperative findings consistent with a successful DCR (ostium spanning the entirety of the sac with brisk drainage into the nasal space). Abnormal DCG findings were categorised into upper or lower canalicular block, common canalicular block, completely closed anastomosis, narrow anastomosis and anastomosis draining into a nasal sinus. Data for each of these categories are summarised in Table 1, with examples of the abnormal DCG images in Figs. 1 and 2.

**Table 1:** Table to show the abnormalities identified from the DCGs of patients who had persistent epiphora or stickiness following DCR surgery

DCG findings	Number of patients (percentage)
Normal postoperative DCR findings with brisk flow of contrast through an open anastomosis into the nasal space	58 (36.3%)
Upper or lower canalicular block	4 (2.5%)
Common canalicular block, with no flow into the sac	13 (8.1%)
Complete surgical anastomosis closure	31 (19.4%)
with a closed sac remnant (Fig. 1c)	20 (12.5%)
with flow though the nasolacrimal duct (Fig. 1d)	11 (6.9%)
Narrow surgical anastomosis	50 (31.3%)
narrow but not high (Fig. 1e)	33 (20.6%)
both narrow and high (Fig. 1f and h)	17 (10.6%)
with retained dye in the erect x-ray suggestive of a retained lacrimal sac (sump syndrome) (Fig. 1g)	19 (11.9%)
Anastomosis into a paranasal sinus (Fig. 2)	3 (1.9%)
Poor quality and unable to be analysed	1 (0.6%)

### Outcomes of patients who underwent further surgery following a failed DCR

**Fig. 3:**
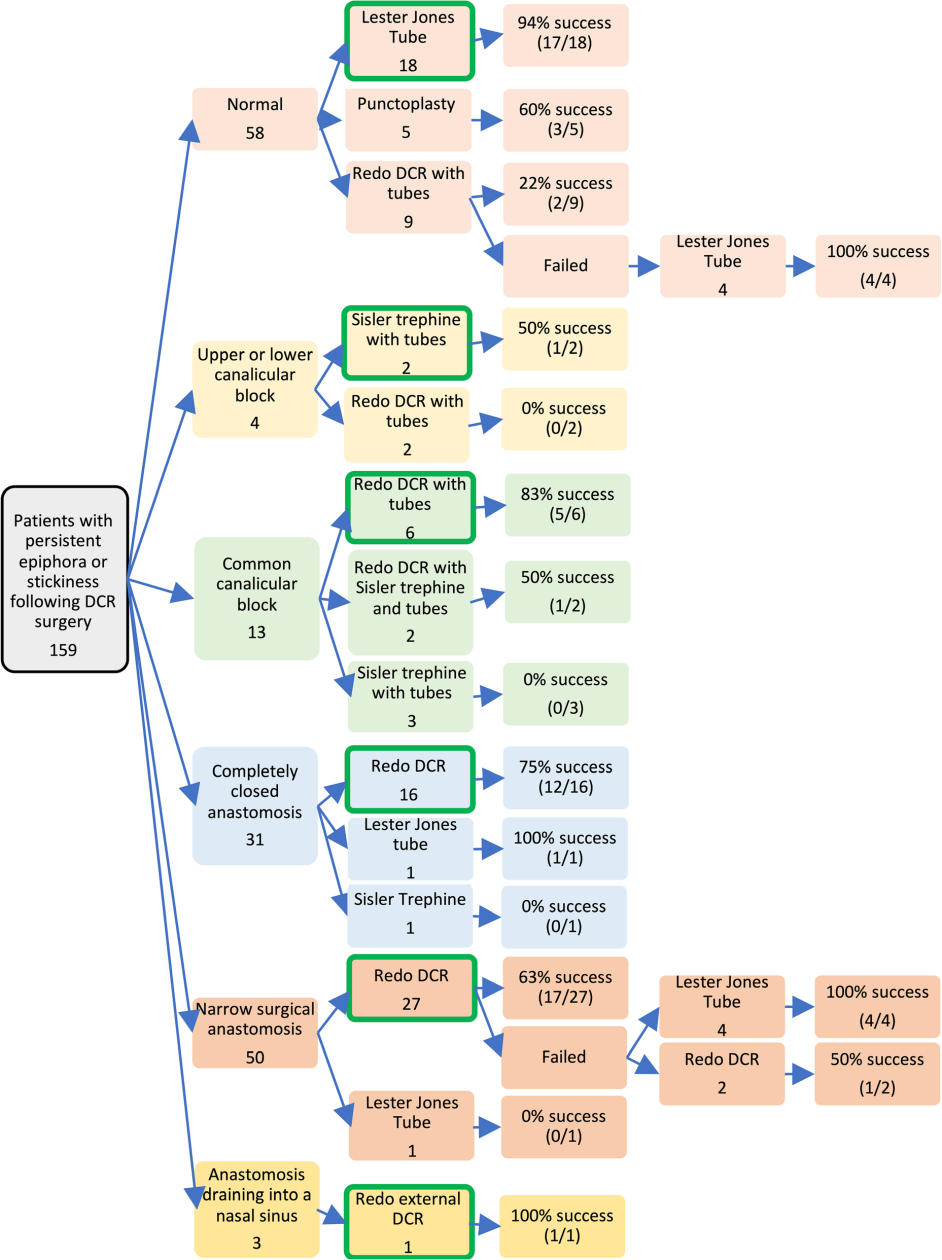
Flowchart to show the success of different surgical categories in each type of DCG abnormality

Of the 159 eyes, 94 (59%) underwent further corrective surgery (Additional file 1). The outcomes of these subsequent surgeries were analyzed according to their DCG-findings category and are summarized in the flowchart (Fig. 3). Tertiary surgeries, if undertaken, are included in the flow diagram.

#### Category 1: Normal post DCR DCG findings

Lester Jones tube (LJT) insertion was the most successful procedure in eyes with a normal DCG, resolving watering in 94% (17/18) of eyes. Redo-DCR was only effective at treating epiphora in 2/9 patient. Four patients in this redo-DCR failed group underwent tertiary LJT with success (4/4 resolution).

#### Upper or lower canalicular block

Sisler trephination with tubes was the most successful procedure in eyes with an upper or lower canalicular block*,* which resolved watering in 50% (1/2).

#### Common canalicular block

Redo-DCR (external or endonasal) with tubes was the most successful procedure in eyes with a common canalicular block with success in 83% (5/6).

#### Completely closed anastomosis

In eyes with completely closed anastomosis, LJT was successful in resolving epiphora in 1 eye (success rate of 100%). However, a much larger number of eyes [11] had redo-DCR (external/endonasal +/− tubes), with resolution of epiphora in 75% (12/16).

#### Narrow anastomosis

Redo-DCR (external/endonasal +/− tubes) was the most successful procedure and successfully resolved epiphora in 17 out of 27 eyes (success rate of 63%).

#### Anastomosis draining into a nasal sinus

Redo-DCR (external + tubes) was the only procedure performed in eyes whose anastomosis drained into a nasal sinus. This was performed in one patient with success (1/1).

### Postoperative follow-up

Patients were followed up for a median of 9.3 months (range 0.7–73.5) after their subsequent surgery.

## Discussion

The surgical options for treating failed DCR surgery are well known, with surgeons tailoring their surgery to the approximate anatomical abnormality. In this study, we examined the DCG anatomical abnormality that led to DCR surgery failure, and reported the success rates of subsequent surgeries according to the anatomical abnormality.

### Causes of epiphora and stickiness recurrence identified on DCG

The most common DCG finding after failed DCR surgery was that of a well-sized and patent anastomosis with brisk flow of contrast into the nasal space (36%), which was followed by a narrow surgical anastomosis (31%), and completely closed anastomosis (19%). This study showed a similar percentage of DCG failure attributed to ‘inadequate ostium size or location’ (84/160, 53%) as that reported 30 years ago at this institution (111/204, 54%) [5]. In that study, Welham et al., also found similar rates of anastomoses into a nasal sinus, reflecting the need for more awareness for this unusual cause of DCR failure. Conversely, we are fortunately now seeing a much lower rate of common canalicular obstruction in our cohort (8%) compared to the past (53%) [5]. It is unclear whether this reflects a difference in the incidence of cicatricial canalicular disease [12] or a reduction in inadvertent iatrogenic canalicular trauma during syringing in clinic, or during intubation in DCR surgery. Unfortunately, as a tertiary referral centre, our department does not have documented canalicular assessment prior to primary DCR surgery, which was performed at other institutions.

### Success rates of subsequent corrective surgery, according to DCG anatomical abnormality

The results of subsequent surgery are presented in the flowchart, with the authors’ recommended surgical option for each DCG category circled in green (Fig. 3). In all but one category, this is the procedure with the highest percentage success rate. In the ‘completely closed anastomosis’ category, despite LJT having 100% success, the authors have recommended redo-DCR surgery in the first instance. This is because only one patient with a narrow surgical anastomosis was treated with LJT. Furthermore, LJT requires life-long maintenance and carries a significant burden to the patient. Redo-DCR showed a reasonable success rate of 75% in a moderate number of eyes [11], and would not alter the success of subsequent LJT insertion, and is thus the recommended secondary procedure in this failure category.

Mitomycin C (MMC) has been used with increased frequency as an adjuvant therapy in DCR surgery. While it has shown to increase surgical success in endonasal DCR revisions [13–15], this same benefit was not found in external approach redo-DCR [16], and at our institution MMC is not routinely used in these cases, and thus is not included in the flowchart.

### Success rates of redo-DCR surgery

Revision-DCR was successful overall in 61% (37/61) for all causes. However, when evaluated based on DCG findings of anatomical failure, rates of success ranged widely from 0 to 100%. When patients are weighing the risks vs. benefits of future surgery, the difference between a 0% or 100% predicted success rate becomes extremely important.

Revision-DCR was least effective in patients with either upper or lower canalicular blockage (0% success) or a normal post-DCR DCG (22% symptom resolution). In patients with normal post-DCR DCG, this poor success rate is likely to reflect the fact that there may be other more proximal points of resistance to flow in the drainage system than at the nasolacrimal duct/anastomosis level. These patients typically have a high tear film, delayed fluorescein dye disappearance test and normal saline lacrimal irrigation test but delayed passage of fluorescein eye drops into the nasal space seen on endoscopy. These patients are described as having a poor lacrimal pump and likely have an unidentifiable (or identifiable abnormality not amenable to surgical correction) of the eyelid, punctum, canaliculus (such as the ‘atonic canaliculus syndrome’) or medial canthal tendon [11]. In the authors department, these patients are offered Lester Jones Tube placement if symptomatic epiphora persists despite conservative management involving regular lid cleaning, hot massage, lubricants, and a 4-week course of topical steroids and chloramphenicol ointment.

Previous reports of symptomatic improvement after external redo-DCR range from 78% [6] to 85% [5]. As previously discussed, the case mix of these groups influences their success rates and makes comparison with this study’s success rate challenging. Ari et al., reported that redo-DCR had 78% success in patients with recurrent dacryocystitis [6], which presumably was due to complete anastomosis obstruction. Their group of patients is equivalent to the cohort in this study with a complete anastomosis closure, who indeed had similar rates of success after redo-DCR (75%). Welham et al., found a higher rate of success post redo-DCR (85%) [5] in failed DCR cases than this study. It is not clear what proportion, if any, of their patients would have had a DCG consistent with a “normal anastomosis”. We have clearly demonstrated that this group of patients had the lowest success rate after revision DCR surgery. Indeed, these are the patients that surgeons are most reluctant to operate on due to low probable success, which can now be quantified for the patient by reference to this study.

### Anastomoses draining into a nasal sinus

Three eyes (1.9%) of 3 patients had their lacrimal sac anastomosed to a nasal sinus rather than the nasal cavity. Unsurprisingly, all 3 patients had external approach DCR, and no tube stents were inserted in 2 cases. Nasal sinus anatomy is highly variable [17]. In particular, agar nasi cells (large, anterior ethmoid air cells) are often encountered during DCR surgery. Entering these air cells can be misinterpreted as entering the nasal space, as is the case in Fig. 2b. Haller Cells are sinuses inferior to the ethmoid air cells, which extend into the roof of the maxillary sinus. They can drain into either the anterior or posterior ethmoidal sinuses and occur in approximately 20% of people [17]. In two cases, the lacrimal sac appeared to drain into Haller Cells (Fig. 2a, c). These particular findings demonstrate how a DCG can be useful in planning revision lacrimal surgery by directing the surgeon to create a new anastomosis rather than erroneously enlarging the pre-existing one.

### Decision making tool

Medicine is now entering into an era of shared decision making with the use of decision-making tools for patients. For this to be viable, patients need to be presented evidence for and against treatment options in a way that is easy for them to understand and compare. This flowchart (Fig. 3) will serve as an invaluable visual guide of surgical success rates and thereby facilitate planning for individualised management.

### Benefits of DCGs

Although the cause of DCR failure can often be determined by careful clinical examination and nasal endoscopy, DCGs can identify precise structural abnormalities that would not be recognised through lacrimal irrigation alone (Table 2). In this study, it identified flow into a nasal sinus, which would only show as partial or complete reflux on lacrimal irrigation. Furthermore, the classic identification of a hard or soft stop on canalicular probing is absent once bone has been removed during DCR surgery. During lacrimal irrigation, the absence or presence of yellow fluorescein in the regurgitated fluid is dependent on having a large enough residual sac. Therefore, sometimes a common canalicular block and a completely closed anastomosis (with no mucus) are confused without a DCG. Additionally, DCGs can reveal ‘sump’ syndrome or ‘birdbox’ anastomosis with hold up of dye in the sac on the erect x-ray, which is not identifiable on lacrimal irrigation alone.

**Table 2:** Table to show the DCG abnormality categories alongside the lacrimal syringing categories and potential misinterpretations of using lacrimal syringing alone

DCG abnormality categories	Lacrimal syringing finding(s) associated with each DCG abnormality category	Abnormalities misinterpreted when using lacrimal syringing alone
Normal	Normal	This syringing category would miss a lacrimal sac diverticulum, a high anastomosis or lacrimal sump syndrome.
Upper or lower canalicular block	Full reflux through the same punctum as the canula is inserted into	Not applicable.
Common canalicular block	Full reflux through upper or both puncta, no fluorescein regurgitation	This syringing category would encompass a very small fibrosed sac remnant, which is unable to hold a significant amount of fluorescein but has a greater rate of success following revision DCR.
Completely closed anastomosis	Full reflux through upper or both puncta, with fluorescein regurgitation	This syringing category would miss a completely closed anastomosis with a partially patent NLD and could encompass an anastomosis into a nasal sinus.
Narrow surgical anastomosis	Partial reflux of saline, partial passage of saline to the nose	This syringing result could encompass an anastomosis into a nasal sinus, and a completely closed anastomosis with a partially patent NLD. These two abnormalities would be suggested with an absence of fluorescein in the nasal cavity on endoscopy, although it would not discern between the two as the nasal endoscope cannot be passed under the inferior turbinate in clinic.
Anastomosis draining into a nasal sinus	Partial or complete reflux on lacrimal irrigation with no passage of saline into the nasal cavity	Syringing alone would only show partial or complete reflux on lacrimal irrigation. DCG is required to identify location of blockage into a nasal sinus.

Although DCGs carry radiation exposure, only 0.0011 to 0.0046Gy [18] is delivered to the lens. In contrast, detectable lens opacities occur from over 100 times this level, at 0.5-2Gy and cataracts occur from 5Gy [19].

### Limitations

The authors’ department benefits from access to walk-in Fluoroscopy for DCGs, facilitating same-day clinical assessment, DCG and discussion of results with the patient. However, we appreciate that this is not available in all clinical settings. Additionally, ordering a DCG means the cost of further appointment(s) as well as a delay in surgical planning. This may deter clinicians from using DCGs regularly. However, to a certain degree, surgeons and patients can still benefit from using this flowchart by substituting the DCG findings with clinical anatomical findings based on lacrimal syringing and nasal endoscopy (Table 2).

The small numbers in some of the categories make statistical comparisons limited and further studies with greater numbers will be needed to confirm these surgical success rates.

### Summary

The management of failure after DCR surgery represents a complex challenge. The structural abnormalities contributing to failure can be identified with DCG imaging. This is the first study to report success rates of redo-DCR surgery according to anatomical findings as confirmed by DCG. These results will help clinicians recommend procedures that are most likely to be successful for their patient’s specific anatomical abnormality, which can be determined by DCG or through a combination of lacrimal syringing and nasal endoscopy. The flow diagram is an easy to interpret visual tool for both physicians and patients, which can be used during a consultation, providing numerical evidence for the shared decision-making process.

## Supplementary Information

**Additional file 1.**

## Data Availability

All data generated or analysed during this study are included in this published article and its supplementary information files.

## Abbreviations

DCG: Dacryocystography
DCR: Dacryocystorhinostomy
NLDO: Nasolacrimal duct obstruction
LJT: Lester Jones Tube
